# Practitioners, and the Marriage of Syphilitics

**Published:** 1919-07-05

**Authors:** C. O. Hawthorne


### PRACTITIONERS, AND THE MARRIAGE OF SYPHILITICS.

**BY C. O. HAWTHORNE, M.D.**

Your leader writer tells us (June 28, page 313) that the "considerations" advanced in my comments of June 21 were "present in his mind" when he penned his first article. Yet even now, and in spite of my invitation, he has nothing to say relative to the meaning he proposes to attach to the phrase "suffering from venereal disease," although in this phrase is lodged the vital point of his policy. When the condition so named exists a medical practitioner, such is your contributor's demand, ought to break the good faith he has pledged to his patient should he learn through some channel or another that his patient is intending matrimony. Here is prescribed a serious rule of conduct, but it is prescribed in terms of uncertain import, and hence as an ethical maxim is defective in practical value. The writer who wishes to enforce such a rule should not be content to have its meaning "present in his mind"; on the contrary, he is morally bound to provide a definition for the assistance and direction of those to whom he offers himself as an ethical guide. He has been asked for this definition. But he makes no reply.

A second "consideration" in my criticism was that if the confidences of patients ought to be betrayed to prevent the spread of venereal disease by marriage, a similar procedure must surely apply to such diseases as tubercle, epilepsy, insanity, and the rest; and I asked how, having granted the one responsibility, the medical profession could consistently decline the other. Your contributor is content to classify this "consideration" as an "obvious" one and to leave his disciples with this very slender measure of assistance.

On the main question I must remark it is he who is making new proposals, not I. My purpose is merely to point out that the protection for which he proposes to pay "a high price" can be readily obtained by anyone who desires it, and this without any invasion of the "honourable tradition" of the medical profession. It is to add what your contributor, I am sure, will agree is "sufficiently obvious" to say that were a certificate of sound health expected as a usual preliminary to marriage there would be nothing invidious in asking for it in any individual instance; and also, that in such circumstances any person contemplating matrimony would doubtless make himself secure on the point of the certificate before advancing to a definite proposal. The suggestion that such a certificate, or the refusal of it, involves a breach of professional secrecy can hardly be taken seriously. Where there is no covenant there can be no breach of covenant, and it is idle to propose that a doctor who on request gives to his patient (free to use or destroy it as he will) a written statement of the results of his professional examination is acting on the same ethical level as one who, having received his patient under the implied covenant of the consulting-room, proceeds later to break this covenant and to act the part of a common informer.

It must further be noted that, tested as a practical measure, your contributor's scheme could prove effective only in very exceptional circumstances. Some persons are honestly unaware of the fact that they are "suffering from venereal disease," and marry without any opportunity for medical intervention; and those who know themselves to be victims of these diseases, and yet in spite of this knowledge intend matrimony, do not invariably announce their intentions to their medical adviser, and still less do they convey to him the name and address of the "prospective spouse." Yet without these various items of information the doctor, however anxious he may be to enlist under your contributor's banner, would be helpless. Hence it could be only in a very occasional instance that a practitioner would have the opportunity of hastening to the protection of the intended partner, unless we are to learn that it is the duty of every practitioner to scrutinise the lists of those between whom there is a "purpose of marriage," as these are announced in his own and neighbouring parishes, and with a view to discover whether, after an examination of his own professional notebooks, any name so announced is that of a person who, to his knowledge, is "suffering from venereal disease." From such an extension of professional obligation I feel sure even your contributor would shrink. Yet unless by an arrangement of this order the practice he advocates could rarely be brought into effective service. It may be reasonable to pay a "high price" for protection if by no other means protection can be secured. But to be asked to pay such a price unnecessarily, and in face of the high probability that the goods will not be delivered, is not an attractive invitation, and I shall be surprised if the medical profession responds to it.

